# Insertional Mutagenesis by CRISPR/Cas9 Ribonucleoprotein Gene Editing in Cells Targeted for Point Mutation Repair Directed by Short Single-Stranded DNA Oligonucleotides

**DOI:** 10.1371/journal.pone.0169350

**Published:** 2017-01-04

**Authors:** Natalia Rivera-Torres, Kelly Banas, Pawel Bialk, Kevin M. Bloh, Eric B. Kmiec

**Affiliations:** 1 Gene Editing Institute, Helen F. Graham Cancer Center and Research Institute, Newark, Delaware, United States of America; 2 Department of Medical Sciences University of Delaware, Newark, Delaware, United States of America; 3 Nemours Center for Childhood Cancer Research, Alfred I. duPont Hospital for Children, Wilmington, Delaware, United States of America; New England Biolabs Inc, UNITED STATES

## Abstract

CRISPR/Cas9 and single-stranded DNA oligonucleotides (ssODNs) have been used to direct the repair of a single base mutation in human genes. Here, we examine a method designed to increase the precision of RNA guided genome editing in human cells by utilizing a CRISPR/Cas9 ribonucleoprotein (RNP) complex to initiate DNA cleavage. The RNP is assembled *in vitro* and induces a double stranded break at a specific site surrounding the mutant base designated for correction by the ssODN. We use an integrated mutant eGFP gene, bearing a single base change rendering the expressed protein nonfunctional, as a single copy target in HCT 116 cells. We observe significant gene correction activity of the mutant base, promoted by the RNP and single-stranded DNA oligonucleotide with validation through genotypic and phenotypic readout. We demonstrate that all individual components must be present to obtain successful gene editing. Importantly, we examine the genotype of individually sorted corrected and uncorrected clonally expanded cell populations for the mutagenic footprint left by the action of these gene editing tools. While the DNA sequence of the corrected population is exact with no adjacent sequence modification, the uncorrected population exhibits heterogeneous mutagenicity with a wide variety of deletions and insertions surrounding the target site. We designate this type of DNA aberration as on-site mutagenicity. Analyses of two clonal populations bearing specific DNA insertions surrounding the target site, indicate that point mutation repair has occurred at the level of the gene. The phenotype, however, is not rescued because a section of the single-stranded oligonucleotide has been inserted altering the reading frame and generating truncated proteins. These data illustrate the importance of analysing mutagenicity in uncorrected cells. Our results also form the basis of a simple model for point mutation repair directed by a short single-stranded DNA oligonucleotides and CRISPR/Cas9 ribonucleoprotein complex.

## Introduction

Single-stranded DNA oligonucleotides (ssODNs) can act as templates for the repair of point mutations in human cells. These molecules direct nucleotide exchange at precise positions and without detectable off target effects [1,2]. While there is great utility in single agent gene editing, the frequency with which single base repair takes place has been consistently lower than needed for long-term development. The mechanism and regulation of single agent gene editing, however, has been elucidated [3–5] and based on these studies two important enhancers of the frequency have been uncovered. The first involves double strand DNA breakage induced by the activity of anticancer drugs such as Camptothecin or VP16, etc in a process that leads to the activation of pathways involved in DNA damage response [6–9]. The second method of increasing the frequency of point mutation repair involves the modulation of the cell cycle. It has been widely reported that synchronization of cells at the G1/S border followed by release, generates a population of cells that are more amenable to gene repair thereby increasing correction frequency by 5 to 10 fold [10–13].

Recently, several research groups have demonstrated that RNA guided engineered nucleases (RGENs) particularly CRISPR/Cas9 systems, can elevate the frequency of point mutation repair when used in combination with single-stranded DNA oligonucleotides [14,15]. By and large, the mechanism and regulation of combinatorial gene editing are similar to the pathways described for single agent gene editing, enhanced by the manipulation of the cell cycle prior to targeting. While this approach has generated a considerable and understandable level of excitement in the field, there are concerns that CRISPR/Cas9 activity, dependent upon or independent from ssODNs, could result in off-site or onsite mutagenesis as a function of its normal mechanism of action [16]. Since CRISPR/Cas9 induces a double strand break that then becomes the template for nonhomologous end joining, it is likely that a heterogeneous population of chromosomal ends is created in corrected and uncorrected cells, particularly at the target site. Intense effort is being placed on developing CRISPR/Cas9 variants that inherently reduces the capacity to target off-site [17–20]. Since the active complex of CRISPR/Cas9 consists of RNA and protein, one approach is to target cells with a pre-formed Ribonucloprotein (RNP) complex that due to a shorter half-life within the cell, may exhibit nonspecific mutagenesis [20–26].

While analysis of off-site mutagenesis occupies the attention of a majority of workers in the field, some reports have focused on mutagenesis at the target site [16,27]. Recently, our laboratory analyzed a population of cells bearing a single base change induced by the combination of CRISPR/Cas9 and ssODNs for altered DNA sequence of the beta globin gene [28]. Our findings indicate that point mutation repair directed by these gene editing tools leave a mutagenic footprint. We found that both insertions and deletions accompany single base repair as judged by allelic analysis of clonally expanded cell populations. These results prompted us to investigate the type of DNA heterogeneity created at the site of single base repair in both corrected and uncorrected cell populations in more detail. To do so, we employed a well-established human cell model system containing a mutant eGFP gene that upon correction enables a simple phenotypic readout that can be confirmed by DNA analysis [29,30]. The mutant eGFP contains a single point mutation that switches a codon for tyrosine (TAC) to a stop codon (TAG). Correction of the stop codon reestablishes the tyrosine codon and rescues the phenotype generating functional eGFP that can be readily measured by FACS. Because this system has been used widely over the course of 15 years to validate and elucidate the mechanism and regulation of gene editing in mammalian cells, we had confidence in its usefulness and robustness to examine point mutation repair and the associated collateral damage created in corrected and uncorrected cells. We isolated targeted cells and expanded clonal populations for DNA sequence analysis. We find that cells bearing a corrected eGFP gene exhibit no collateral damage and no onsite mutagenesis. In contrast, some of the expanded clones from populations of targeted cells in which no phenotypic change was observed, exhibited intact mutant sequence without associated modifications, while others exhibited a wide range of indel formation, including insertional mutagenesis in the creation of hybrid genes encoding truncated proteins. Our results provide the basis for a new model of gene editing for point mutations and emphasize the importance of evaluating all cells targeted for gene editing by CRISPR/Cas9 and ssODNs, especially as gene editing extends toward human therapy.

## Results

**Fig 1:**
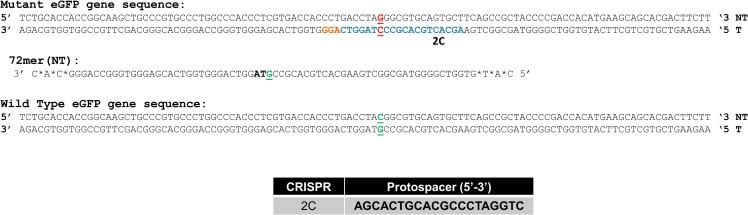
Model system for gene editing of the mutant eGFP gene. The appropriate segments of the wild-type and mutated eGFP gene with the targeted codon, located in the center of the sequence, are displayed in green and red. The nucleotide targeted for exchange is bolded and underlined. The highlighted bases in blue represent the 2C CRISPR protospacer sequence and the orange bases highlight the PAM site. The oligonucleotide used in these experiments is 72 bases in length bearing phosphorothioate modified linkages at the three terminal bases; the 72-mer targets the non- transcribed (NT) strand (72NT).

Fig 1 displays part of the sequence of the mutant eGFP gene that has been inserted as a single copy into HCT 116 cells and driven by a CMV promoter [29] to generate the cell line HCT 116–19. The point mutation target is the terminal base of the TAG stop codon highlighted in red. Gene editing using CRISPR/Cas9 and ssODNs aims to rescue the mutation, converting the G base to a C and restoring the normal tyrosine codon (TAC). The wild type eGFP sequence is depicted as is the sequence of the 72 base single-stranded oligonucleotide, which is complementary to the non-transcribed (non-template) strand (72NT). Also included in the figure is the sequence of the protospacer and the PAM sequence highlighted in blue and orange respectively as well as the the sequence of the mutant gene. This specific combination of CRISPR/Cas9 and ssODN has been shown previously to be optimal for RGEN-directed correction [14]. In the experiments reported herein, we utilize the 72NT oligonucleotide since previous data have established that targeting the non-transcribed strand at this ssODN length leads to a higher level of gene editing [31]. In addition, the use of the complementary oligonucleotide, targeting the transcribed strand, leads to artefactual annealing to the sgRNA component of the CRISPR/Cas9 complex, reducing overall activity [14]. Successful correction of the point mutation leads to the production of a functional eGFP which can be detected and quantified by FACS.

**Fig 2:**
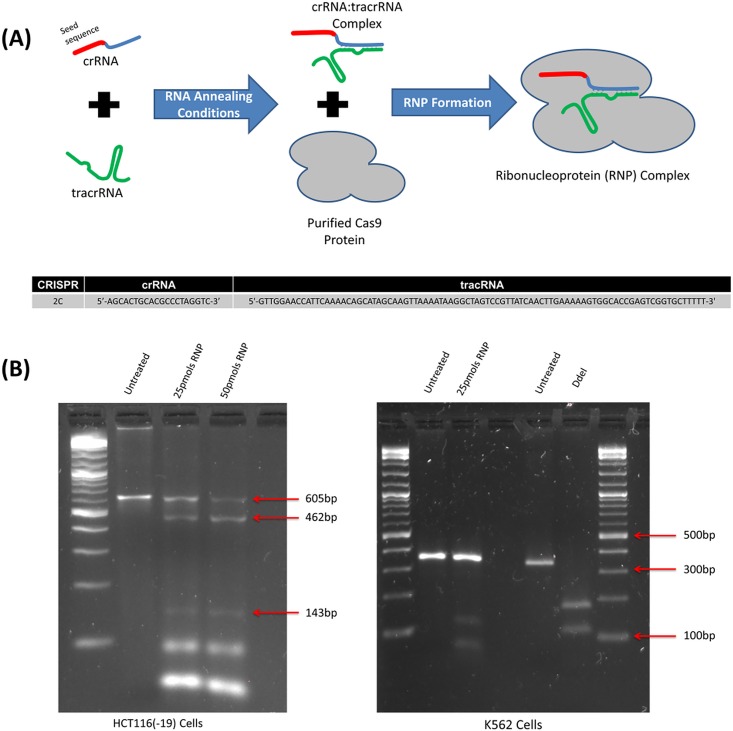
**(A) CRISPR/Cas9 Ribonucleoprotein Assembly Reaction.** crRNA provides target specificity (20 bases, red section) corresponding to the 2C protospacer sequence and an interaction domain (blue) with the tracrRNA (green). crRNA and tracrRNA are annealed in equimolar concentrations. Cas9 protein (gray) is added to complete RNP assembly. Guide RNAs (gRNAs) direct and activate the Cas9 endonuclease which then cleaves the target DNA. The lower section of the figure shows the 2C seed sequence and the tracrRNA sequence. **(B) *In vitro* RNP Digestion.** Genomic DNA was isolated from untreated HCT 116–19 cells and PCR used to generate an amplicon of size 605bp, which surrounds the sequence of the integrated mutant eGFP gene. The amplicon was combined with 25pmols and 50pmols of RNP complex respectively and incubated for 40 minutes at 37°C. In the complete reaction, two products were generated with sizes consistent with fragments predicted from the specific cut site designed for the RNP complex. As a control, the RNP complex was incubated with an amplicon generated from the HBB gene 345 base pairs in length from cell line K562. A control digest was performed on the 345 base amplicon with the restriction enzyme DdeI.

In previous studies, both components (sgRNA and Cas9) were generated from a plasmid expression vector [14]. In this study, however, we provide the CRISPR/Cas9 as a ribonucleoprotein complex that is preassembled prior to introduction into the cells. Fig 2 provides a schematic of our assembly process. The crRNA and the tracrRNA are identical in sequence to the longer sgRNA used previously although they are used as separate RNA molecules in this protocol. The crRNA and tracrRNA are reannealed by mixing RNA oligos (crRNA and tracrRNA) in equimolar concentrations with subsequent addition of purified Cas9 protein. To measure inherent activity of the RNP with regard to its capacity to cleave DNA, we carried out an *in vitro* reaction wherein we assessed the capacity of this particular RNP complex to induce double strand DNA cleavage in a specific fragment of DNA. The fragment was created by PCR amplification across the mutant eGFP target site generating a 605 base pair template containing the target site for the RNP. The preassembled RNP was mixed with this fragment at various concentrations for 40 minutes followed by deproteinization by Proteinase K. The digestion fragments were visualized after gel electrophoresis and the data are presented in Fig 2B. As predicted, the RNP efficiently catalyzes double strand DNA cleavage of the specific fragment but not of a fragment lacking the target site. These results support the notion that the RNP complex assembled under our reaction conditions contains the appropriate level of activity and specificity for inducing double strand DNA cleavage.

**Fig 3:**
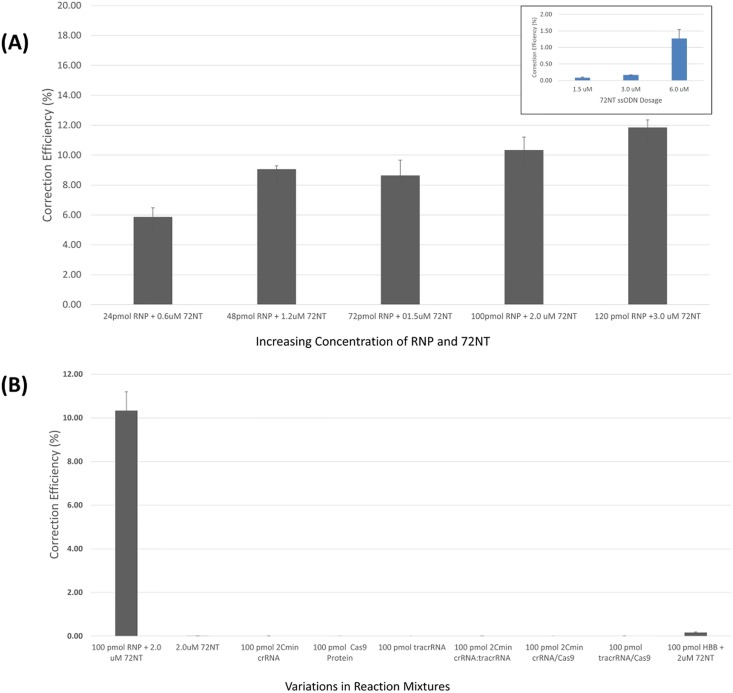
**(A) Gene editing is dose dependent when directed by the RNP and the ssODN.** Synchronized and released HCT 116–19 cells were electroporated with 24–120 pmol CRISPR/Cas9 RNP and 0.6–3.0 μM of 72mer. After a 72-hour recovery period, gene editing activity was measured using a FACSAria II flow cytometer. Gene editing is displayed as correction efficiency (%), determined by the number of viable eGFP positive cells divided by the total number of viable cells in the population. Each treatment was performed in triplicate and standard error is illustrated with accompanying bars. **Inset: Single agent gene editing**. Gene editing activity directed by the single-stranded oligonucleotide (72NT) in the absence of the RNP complex under identical conditions is presented as a function of increasing concentration. **(B) Gene editing activity is dependent on all components being present in the reaction mixture.** Synchronized and released HCT 116–19 cells were electroporated with 100pmol of the crRNA, Cas9 Protein, tracrRNA and 2.0 μM of the 72NT, as a complete reaction. Identical mixtures, lacking the indicated reaction component, were carried out in parallel. In one specific reaction mixture, the RNP specific for the beta globin gene replaced the RNP specific for the eGFP gene (far right bar). After a 72 hour recovery period, gene editing activity was measured using a FACSAria II flow cytometer. Gene editing is displayed as correction efficiency (%), determined by the number of viable eGFP positive cells divided by the total number of viable cells in the population. Each treatment was performed in triplicate and standard error is illustrated with accompanying bars.

For the cell-based gene editing reaction, the RNP was combined with 72NT at a prescribed molar ratio of 1:2.5 and immediately electroporated into HCT 116–19 cells. Cells are allowed to incubate for 72 hours after which time they are processed for FACS analysis. We carried out a dose curve with increasing concentrations of the preassembled RNP, while maintaining a constant ratio of ssODN (72NT) to RNP. The data are presented in Fig 3A and exhibit a steady increase in correction efficiency, rising steadily from the initial level of 24 pmols of RNP to a high level when 120 pmols are used in the reaction. In contrast, single agent gene editing using only the 72NT produces a much lower level of gene editing, as previously reported [1,2]. These data suggest that the RNP particle used in combination with the 72NT oligonucleotide can promote gene correction at a level approaching 10 to 12% reproducibly. The next experiment addresses the question of the importance of each reaction component. We chose to utilize a complete reaction mixture containing 100pmol of RNP complex and 2.0μM of 72NT respectively. As shown in Fig 3B, elimination of one or two of the essential reaction components eliminates gene editing activity completely. In addition, we tested a complete reaction mixture for activity after replacing the specific RNP complex with one that targets the beta globin gene. No reproducible levels of gene editing were observed emphasizing the requirement for the specific RNP particle coupled to the single-stranded oligonucleotide to direct correction of the point mutation in the eGFP gene. The reaction mixture containing all of the relevant components, however, promotes correction efficiency of approximately 10%, consistent with the previous data (Fig 3A). This level of gene editing directed by the RNP/72NT complex can therefore produce a sufficient level of eGFP positive cells separated from eGFP negative cells to enable robust single cell sorting by FACS and subsequent clonal expansion.

**Fig 4:**
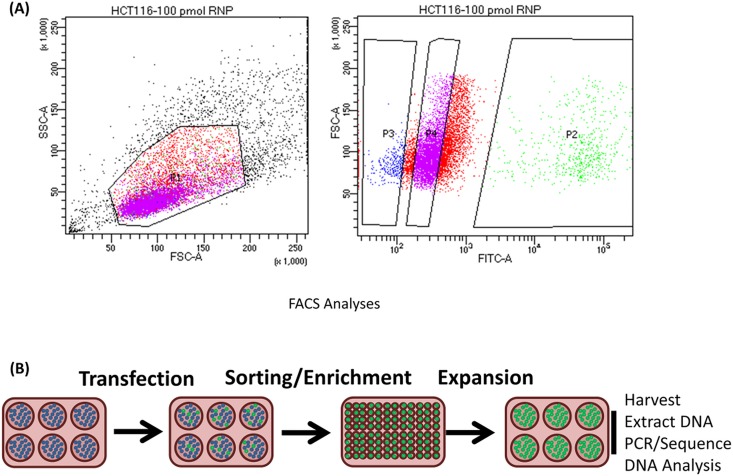
**(A) FACSAria II plots of gene editing activity in HCT 116–19 cells**. HCT 116–19 cells synchronized for 24 hours at the G1/S border and released were electroporated with 100 pmol of RNP complex and 2.0 μM of the 72NT ssODN. After 72 hours, the cells were analyzed using FACS and single cells were sorted individually into 96-well plates. Two distinct populations were collected. The population of live, green cells (labeled as P2 on the FACS plot) as well as the population of live, non-green cells (labeled as P3) were segregated into separate clonal expansion plates. **(B) Experimental strategy isolation of single cell clones.** Cells exhibiting eGFP expression were scored positive and sorted using a FACSAria II flow cytometer as single cells into individual wells for clonal expansion. Cells lacking eGFP expression isolated and sorted in a similar fashion and expanded under the same conditions. DNA was then isolated and the eGFP gene was amplified and subjected to Sanger sequencing to analyze gene editing activity surrounding the target site.

Fig 4A presents a side scatter plot of a complete gene editing reaction on a population of HCT 116–19 cells. The segmented plot illustrates a distinct percentage of cells in the P2 quadrant, representing eGFP positive cells that can be distributed as individual cells into a single well of a 96 well plate. In a similar fashion, uncorrected cells from the population displayed in quadrant P3 can also be isolated. Fig 4B displays the experimental flow following transfection and sorting, enrichment and finally clonal expansion prior to DNA harvesting, extraction and sequence analysis. Using this experimental strategy, we are able to interrogate the allelic composition of corrected and uncorrected single cells specifically measuring the degree of DNA heterogeneity, or onsite mutagenesis, accompanying successful and unsuccessful gene editing activity.

**Fig 5:**
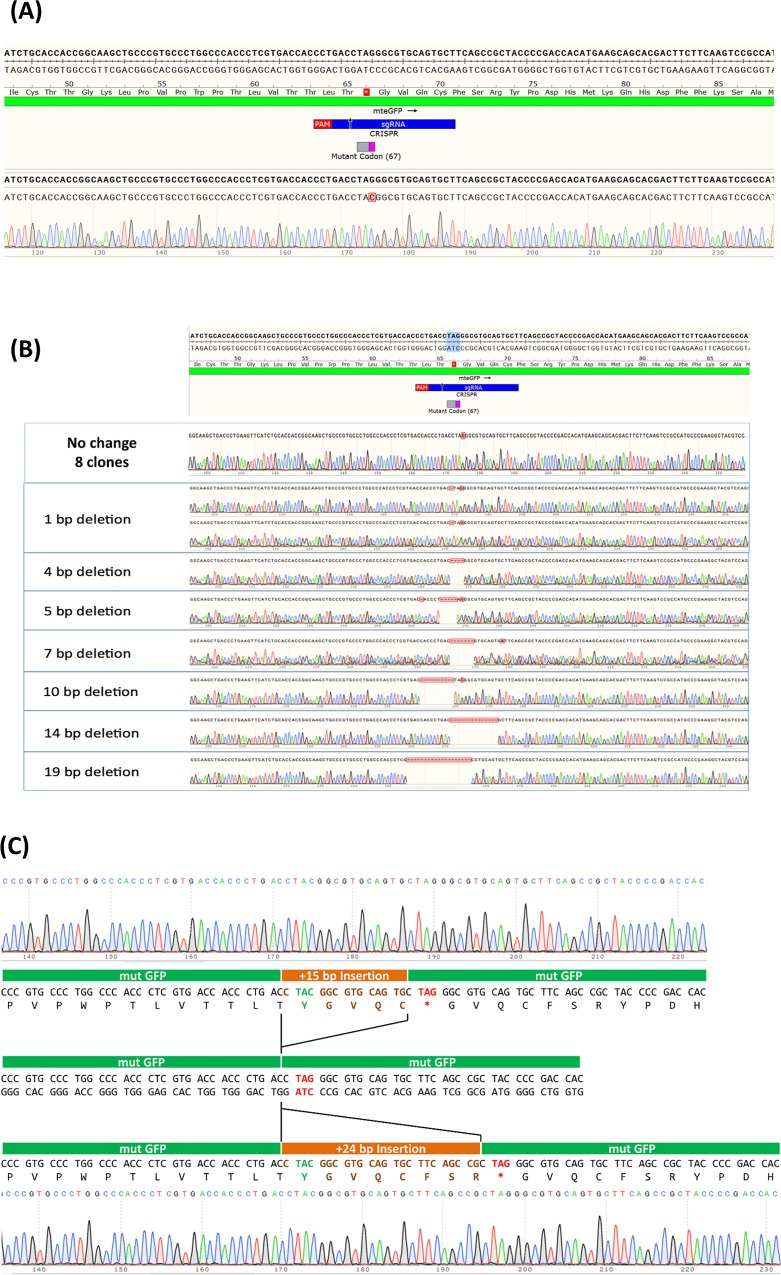
**(A) Allelic analysis of eGFP positive cells expanded as a clonal population.** Clonally isolated and expanded eGFP positive samples (sixteen clones) were analyzed at the site surrounding the targeted base and DNA from each, harvested, purified, amplified and sequenced. Allelic analysis was carried out using Sanger sequencing, assembled using SnapGene and compared to the sequence of a wild-type allele which is illustrated at the top of the figure; the cut site of the RNP complex is indicated as a small black arrow located on the green bar (2C crRNA). **(B) Allelic analysis of eGFP negative cells expanded as a clonal population.** Fifteen individual samples, expanded from cloned originating from the uncorrected population were randomly selected and analyzed for indel formation at the site surrounding the target nucleotide. As above, allelic analysis was carried out using Sanger sequencing and assembled SnapGene. Once again, the sequence of a wild-type allele at the top of the figure along with the cut site of the RNP is presented. **(C) Allelic analysis of eGFP negative cells presenting insertions.** Two individual clones from the uncorrected population displayed insertions of 15bp (top panel) and 24bp (bottom panel), respectively. The center panel represents the mutant eGFP gene sequence with the mutant codon in red. The inserted bases are highlighted in orange with the corrected tyrosine codon depicted in green and the mutant stop codon represented by a red asterisk. The boundaries of the insertions are denoted by black bars.

After sorting, isolation and expansion of corrected and uncorrected single cells, the DNA sequence of multiple clones was analyzed using direct Sanger sequencing following PCR amplification of a 718bp long PCR fragment. As shown in Fig 5A, precise conversion of the TAG codon, to TAC, (light blue highlighted area) confirms phenotypic expression in the eGFP positive clones, at the DNA level. Sixteen eGFP positive clones were expanded in the same fashion and *all* contained the converted DNA sequence as presented in Fig 5A. No sequence alterations or onsite mutagenesis was observed within the 718 base pair DNA region in these experiments. The DNA sequence readout found in all the clones is provided in the lower half of the figure to show that no contaminating or background sequence is present, indicating that single cell clonal expansion from the corrected population was successful. Fig 5B illustrates the genotypic analyses of 17 eGFP-negative clonally expanded cells, selected at random from the sorted, uncorrected population. In almost half of these clones, the TAG codon remains intact and no sequence variation is observed within the region examined. In approximately half of the other clones examined on-site onsite mutagenesis consisting of both deletion and insertion mutations of varying lengths. This mutagenic activity ranged from a one base pair deletion surrounding the target site to a 19 pair deletion to a 24 base pair insertion immediately downstream from the targeted base. These results demonstrate that onsite mutagenesis occurs during RNP/ssODN gene editing reactions in cells that fail to achieve the desired phenotype.

**Fig 6:**
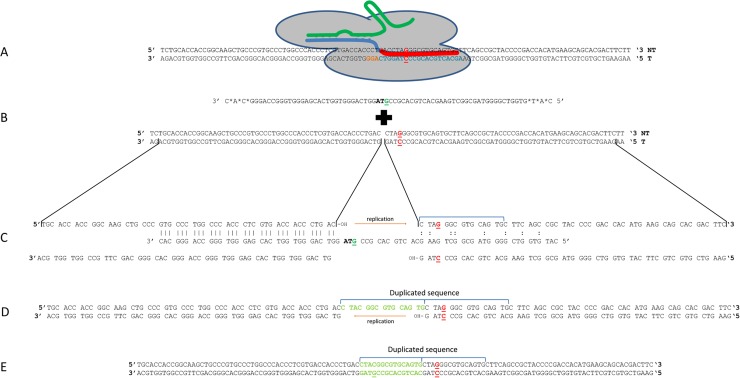
A model for point mutation repair directed by an RNP complex and a short single-stranded DNA oligonucleotide. (Panels A and B); the RNP particle induces a double strand break at the target site generating two free 3’ hydroxyl ends on each strand of the broken DNA. (Panel C); the oligonucleotide aligns in imperfect homologous register with the non-transcribed strand of the chromosome. The DNA replication machinery fills the gap starting from the 3’ hydroxyl end and completing by ligation to the 5’ phosphate at the opposite side of the gap. The single-stranded oligonucleotide serves as a template for the replication process. (Panels D/E), dissociation of the single-stranded oligonucleotide allows for the newly synthesized DNA to act as a template for DNA replication in the opposite direction on the bottom strand followed by ligation.

We examined the genotype of the two clones containing DNA insertions, neither of which exhibit a change in phenotype as judged by the absence of green fluorescence. These two clones are instructive not only for what they tell us about the potential for on-site mutagenesis but also what they tell us about a mechanism for DNA insertion driven by single-stranded DNA (see Discussion). In Fig 5C, we provide the DNA sequence of the uncorrected clone containing a 15 base pair insertion. We illustrate how this insertion created a new frameshift generating the corrected TAC tyrosine codon and concomitantly creating a new TAG, stop codon. Thus, this clone appears to have been corrected at the targeted base but that correction is not reflected in a phenotypic change. As such, the insertion expands the gene by five codons. In the same fashion, a second clone containing a 24 base pair segment, inserted at the identical position, is also displayed in Fig 5C. In this case, 8 new codons, preceding the newly created stop codon, have now become inserted. Thus, CRISPR/Cas9 single-stranded oligonucleotide gene editing can generate a novel stretch of amino acids that are not encoded by the targeted gene. The DNA insertion matches, in perfect register, a section of the single-stranded oligonucleotide when placed in this reading frame. Thus, our data suggest that sections of the oligonucleotide can be inserted into the target gene, resulting with the simultaneous correction of the point mutation and the generation of a mutagenic footprint (see Fig 6). The degree of mutagenesis observed in the uncorrected population is broad, signaling the importance of analyzing a representative sample of the entire population of cells targeted for genetic alteration.

## Discussion

Collateral mutagenesis, generated by the action the CRISPR/Cas9 gene editing tool, has been a central focus of both advocates and critics of this technology. Sophisticated molecular cloning approaches to refute, diminish or downplay the degree of off-site mutagenesis have been offered by many of the leading laboratories in the field [20,32,33]. But, in many cases, results rely in large part on proving a negative. In fact, it is debatable as to whether or not off-site mutagenesis can be completely eliminated as a potential side reaction in therapeutic gene editing. More recently, focus has been placed on the potential of *onsite* mutagenesis, an outcome of the normal activity of RGENs. The inherent response of a cell to repair the double strand break through the process of non-homologous end joining is at the core of the current genetic revolution, partially inspired by RGENs, that has made the generation of gene knockouts in many eukaryotic cell types a routine lab procedure. In contrast, onsite mutagenesis becomes more relevant when the objective of the gene editing protocol is not to disable but rather to repair a gene bearing a point mutation, and eventually, to direct seamless insertion of a fragment of donor DNA.

Most of the studies focused on onsite genetic heterogeneity, examine genes for which there is complex readout, as in the case of human stem cells, often requiring drug selection to identify the targeted cells [27]. Primary cells and even transformed cells can respond negatively to selective pressures when challenged with either chemo-toxins or antibiotics. Our laboratory has also been examining onsite mutagenesis as a function of single base gene editing, the repair of a point mutation in human cells [28]. While we too have utilized a native gene, human HBB, to validate our initial findings, we find it essential to employ a reproducible, robust model system that has a long history of validated readout that can measure phenotype, protein function and genotype without exogenous manipulation.

In this paper, we utilize a model system in which a mutated eGFP gene, integrated as a single copy into HCT 116 cells [30], is targeted for repair by the combination of a CRISPR/Cas9 RNP and a specific single-stranded DNA oligonucleotide. Successful conversion of the point mutation transforms a stop codon to a tyrosine codon enabling translation and expression of functional eGFP. Because the cells can be cloned and examined as uniquely expanded populations, allelic analysis of gene editing activity in both corrected and uncorrected populations is simple. We demonstrate that the combination of the RNP complex and a 72-mer directs gene repair of the point mutation in an efficient and reproducible fashion. Keeping the molecular ratio of the RNP and the single-strand DNA oligonucleotide constant but raising the total amount in the reaction induces a dose-dependent response which begins to plateau above 10%; at an 8 to 10 fold higher level than when the ssODN is used as the sole gene editing agent. All of the appropriate reaction components are required for successful point mutation repair and the separation of the corrected and uncorrected cells can be achieved in a straightforward fashion.

Our rationale for using the RNP is that the active components of the CRISPR/Cas9 system will be delivered to the nucleus at approximately the same time facilitating a more constant initialization of the gene editing reaction. Previously, we had used a plasmid expression system in which Cas9 is expressed from the same plasmid as the sgRNA. In our studies, we confirm point mutation repair driven by the combination of the RNP and the ssODN.

With regard to the examination of DNA heterogeneity in corrected and uncorrected populations, we find that cells, identified by FACS as being corrected, exhibit precise single base repair at the target site. In no case did we observe any genetic alteration with cells from the corrected population for a proximal distance of 718 bases. In addition, we did not observe any nucleotide changes that would result in a conservative change in amino acid sequence still enabling expression of alternative wild type eGFP.

The clonal expansion of a population of cells that did not exhibit phenotypic correction generated a panel of genetic alterations ranging from uncorrected, yet intact, to a cell line bearing a 19 base deletion surrounding the target site to the insertion of 24 bases surrounding the target site respectively. Of the 17 clones tested, eight had no change to the mutant DNA sequence, perhaps indicating that the RNP complex had not reached the target site in those cells, had not induced by double strand break at the site or had induced a double strand break which was properly and efficiently repaired with or without the aid of the single-stranded oligonucleotide. We observed a wide range of DNA sequence deletions starting with a single base deletion and ending with a 19 base deletion, heterogeneity that surrounds the nucleotide targeted for gene repair. No other sequence alterations outside of the target site were observed, again within the proximal 718 bases. The one clone harboring a 15 base insertion, appears to arise through a duplication of the adjacent 15 bases located 5’ to the target site, as well as a 24 base insertion that appears to have come from the same DNA source. Our results indicate that onsite mutagenesis clearly occurs in the uncorrected population of cells, exhibiting a wide range of indel formation. We have previously observed a similar phenomenon in a separate series of studies wherein in the objective was to induce a single base change in the genome of K562 cells (28). In that system, however, we were unable to examine the impact on phenotypic changes and thus this model system expands and confirms those studies, demonstrating on-site heterogeneity as a function of gene editing reactions that include single-stranded DNA oligonucleotides.

Our data align with associated studies that examine the insertion of a longer fragment of DNA at a precise site. Merkel et al [27] recently published an elegant study in which indel formation was observed at the target site catalyzed by intact CRISPR/Cas9, as well as associated single or dual Nickases. Taken together, these results expand upon data from earlier work in which the objective was to modify the target site excessively without introducing unwarranted changes [26,32,33]; all of these studies reported site alteration.

Our specific interest herein is not to develop a strategy to insert a large fragment of DNA, but rather to use a short piece of donor DNA, a short single-stranded oligonucleotide, to perform genetic surgery as a way to repair single point mutations. Over the course of the last 15 years we and others have established the mechanism and regulation of short oligo induced gene editing [2–4,10,11,34]. More recent studies have shown that oligonucleotides of length between 49 and 72 bases respectively can direct single base repair as the sole agent of the gene editing reaction or in combination with both TALENs and CRISPR/Cas9 [33,35]. Thus, while the mechanism of action of single base repair, directed by these oligonucleotides of restricted length, may differ from the mechanism by which DNA fragment or gene insertion takes place, it is now apparent that the same type of allelic analysis should be performed on at least a sample of both corrected and uncorrected targeted cells generated from both approaches. It is therefore critical that studies are conducted in reliable, robust and validated testing systems to explore the degree of collateral damage directed by CRISPR/Cas9 to more fully understand the remarkable power of this gene editing tool especially in light of its therapeutic potential.

We did not observe any homologous recombination events from distal sites, wherein genetic information is provided by adjacent chromosomes to aid in the repair of the fragmented DNA. The DNA sequence of the insertion clones, however, enables a continuation of the reading frame through several codons until a stop codon is generated so additional genetic information was proved in some fashion. Both insertion clones contain the exact corrected point mutation but do not score as eGFP^+^ because the inserted DNA creates a stop codon 15 or 24 bases downstream from the targeted nucleotide respectively. This is an interesting example of how double strand DNA breakage can provide a site for DNA insertion of exogenous or repetitive segments as the cell responds to chromosomal damage. These data provide us with insight into the overall mechanism by which short oligonucleotides and the RNP execute the repair of a point mutation in a mammalian cell and enable the development of a model that explains our results. Fig 6 displays a model that we believe explains the generation of cells bearing only a corrected genotype, as well as cells bearing both corrected genotype and phenotype.

A number of sophisticated models have been put forward to explain the insertion of exogenous DNA templates for the repair of single base mutations in gene editing reactions [28,36,37]. These models are based on a process known as Homology Directed Repair (HDR) and likely help explain the results in many of the studies wherein the objective was to insert longer pieces of DNA. In their important study, Paquet et al [38] developed a gene editing methodology, known as CORRECT, for introducing mono- and bi-allelic sequence changes.These workers were successful in elevating the accuracy of HDR through the incorporation of blocking mutations that modify the interaction between CRISPR/Cas9 and the PAM sites resulting in *scarless* genome editing. As a group, these approaches and the models that are generated by them are somewhat complex because they involve specific enzymatic activities and sophisticated reengineering of some of the reaction components.

Our model is much simpler as we have based it on the well-accepted and standard model Double Strand Break Repair [38,39, 40]. When a double strand break occurs in a mammalian chromosome (in the case of gene editing, induced by CRISPR/Cas9 activity), activated exonucleases recognize the break and resect the broken ends to varying degrees, a biochemical reaction that takes place regardless of whether the break is designated for repair through the process of homologous recombination or nonhomologous end joining. In the case of homologous recombination however, usually occurring during S-phase of the cell cycle [41,42], proteins involved in DNA recombinational repair load onto the broken ends. Subsequently, a sister chromatid provides the DNA template to enable the broken strand to once again be made whole through the process of gap filling by DNA replication. Since crossover of one strand of DNA from the sister chromatin provides the template, its original partner strand is displaced and becomes the template for gap filling through DNA replication, albeit in the opposite polarity. Thus, the gap created by the original double strand break is repaired through the utilization of an exogenous piece of DNA that serves as a source of genetic information and the template for replication activity and gap filling. We believe this general concept can help explain the appearance of these two insertional mutants and may also explain previous data including the overall reaction of how single-stranded oligonucleotides direct point mutation repair in mammalian cells [1,2,8,34].

We propose that the RNP particle, as illustrated in Fig 6, Panel A, interacts at the target site and catalyzes a double strand break leaving two 3’ hydroxyl ends available for extension by the DNA replication machinery. Non-homologous end joining activity resects the broken ends and the degree of this resection varies from clone to clone (Panel B). The clones expanded from the uncorrected population support the fact that varying degrees of resection take place (see Fig 5B) because DNA insertions of 15 and 24 baes were found. As illustrated in Panel C, the oligonucleotide (red) pairs stably with the target gene via sequence complementarity, bridging the gap in the top strand. The binding is more stable upstream since the ssODN aligns in homologous register using perfect complementarity. Downstream from the break site, the base pairing must be incomplete because the data reveals a duplication of adjacent sequences. The partial binding downstream from the resected site is, in fact, energetically favorable based on calculations of free energy (approximately ΔG of -2.6). In our system, the oligonucleotide has been designed to be complementary to the non-transcribed strand and thus we can depict the polarity of pairing partners with confidence. As illustrated in Panel D and as a result of resection, a free 3’ hydroxyl end on the top strand is now available for extension by DNA replication. In this simple model, the oligonucleotide acts as a template for the replication machinery to fill in the gap in the upper strand. For these two clones, the single-stranded oligonucleotide used in the gene editing reaction contains a G residue at its center because it is designed to create a single base mismatch with the G residue in the gene and promote mismatch repair. This strategy is based on work on single agent gene editing wherein the objective is not DNA insertion but rather nucleotide exchange through the process of mismatch repair or by incorporation of the oligonucleotide into a growing replication fork (see models in references 1, 2, 3). In contrast to some other models of gene editing [28,36–38], our sequencing data indicate that the oligonucleotide itself does not insert directly because, if this had happened, then the base at that position, identified in the genomic sequence (Fig 5C), would have been a G, not a C. After serving as a template for replication, the oligonucleotide dissociates (Panel D) and DNA replication is initiated on the opposite strand and in the opposite direction by utilizing the free hydroxyl group for extension as illustrated in Panel E. We have termed this variant of gene editing, EXACT, for *EX*cision *A*nd *C*orrective *T*herapy; it may also be the general mechanism by which point mutations are repaired in gene editing reactions as directed by short oligonucleotides and double strand DNA breaks at the target site.

In principle though, our results do align with the conclusions of Schumann et al [26] and others [36,37] in that the activity of the CRISPR/Cas9 system provides a framework for the repair of resected regions of genomic DNA. Importantly, however, we do not observe fragment insertion for point mutation repair because none of the clones examined in this study contained the G nucleotide at the target site. Since double strand DNA breaks are widely recognized as being both dangerous to cell viability and highly recombinogenic, it is likely that multiple pathways are used to regenerate a contiguous chromosome. The mechanism of repair may be dictated by the type and structure of donor DNA available at the site of damage.

## Materials and Methods

### Cell Line and Culture Conditions

HCT 116 cells were acquired from ATCC (American Type Cell Culture, Manassas, VA). The HCT 116–19 was created by integrating a pEGFP-N3 vector (Clontech, Palo Alto, CA) containing a mutated eGFP gene. The mutated eGFP gene has a nonsense mutation at position +67 resulting in a nonfunctional eGFP protein. For these experiments, HCT 116–19 cells were cultured in McCoy’s 5A Modified medium (Thermo Scientific, Pittsburgh, PA) supplemented with 10% fetal bovine serum, 2mM L-Glutamine, and 1% Penicillin/Streptomycin. Cells were maintained at 37°C and 5% CO_2_. The eGFP targeting custom designed 72-mer oligonucleotide was synthesized by IDT (Integrated DNA Technologies, Coralville, IA).

### CRISPR/Cas9 RNP Design and Complexing

The mutant eGFP gene sequence was entered into the Zhang Lab’s online generator (http://crispr.mit.edu/) and the CRISPR guide sequences which binds with close proximity to target (TAG = 0) was chosen. crRNA, tracrRNA and Cas9 protein were kind gifts from Integrated DNA Technologies (Coralville, Iowa) and stored and utilized according to their suggestions. RNP assembly was performed by mixing RNA oligos (crRNA and tracrRNA) in equimolar concentrations to a final duplex concentration of 45μM. For the RNA to duplex the mix was heated at 95°C for 5 minutes and allowed to cool to room temperature (15–25°C). For each sample crRNA:tracrRNA (45μM working solution) and Cas9 protein (60μM stock solution) were diluted in their respective buffers to a final volume of 5μL each to achieve the desired treatment concentration. Prior to mixing with cells crRNA:tracrRNA duplex and Cas9 protein we mixed and set to incubate at room temperature for 15 minutes. The same annealing conditions and reactions were carried out in the assembly of the mutant eGFP or B-globin gene crRNA (28) RNP.

### Experimental Strategy

For all experiments, HCT 116–19 cells were synchronized for 24 hours with Aphidicholin at the G1/S border prior to introducing the Cas9 ribonucleoprotein (RNP) complex or CRISPR/Cas9 generated from an expression construct. The CRISPR expression plasmid was constructed using standard cloning methods following the latest oligo annealing and backbone cloning protocol with single-step digestion-ligation. The CRISPR guide sequences were cloned into the pX330 backbone vector (Addgene plasmid 42230), a human codon-optimized SpCas9 and chimeric guide RNA expression plasmid. Single-stranded DNA oligonucleotides used in this study are 72 base pairs in length and designed as depicted in Fig 1. RNP assembly was performed by mixing RNA oligos (crRNA and tracrRNA) in equimolar concentrations to a final duplex concentration of 45μM. For the RNA to duplex the mix was heated at 95°C for 5 minutes and allowed to cool to room temperature (15–25°C). For each sample crRNA:tracrRNA (45μM working solution) and Cas9 protein (60μM stock solution) were diluted in their respective buffers to a final volume of 5μL each to achieve the desired treatment concentration (24-120pmol). Prior to mixing with cells crRNA:tracrRNA duplex and Cas9 protein we mixed and set to incubate at room temperature for 15 minutes. Electroporation transfection was performed by mixing cells at concentration of 5x10^5^ cells/100 microliters along with the RNP and ssODNs in a 4mm gap cuvette (BioExpress, Kaysville, UT) (250V, LV, 13ms pulse length, 2 pulses, 1s interval) using a Bio-Rad Gene Pulser^TM^ XCell Electroporation System (Bio-Rad Laboratories, Hercules, CA). Cells were then recovered in 6-well plates with complete growth media at 37°C for 72 hours prior to analysis.

### Analysis of Gene Edited Cells and Transfection Efficiency

HCT 116–19 cell fluorescence (eGFP^+^) was measured by a BD FACSAria II (BD Biosciences, San Jose, CA). Cells were harvested by trypsinization, washed once with 1x PBS (-/-) and resuspended in buffer (0.5% BSA, 2mM EDTA, 2μg/mL Propidium Iodide in PBS -/-). Propidium iodide was used to measure cell viability as such, viable cells stain negative for PI (uptake). Correction efficiency was calculated as the percentage of the total live eGFP positive cells over the total live cells in each sample. Error bars are produced from three sets of data points generated over three separate experiments using basic calculations of Standard Error.

### RNP in Vitro Activity

Cellular gDNA was isolated from pellets of 1 x 10^6^ untreated HCT 116–19 cells using Qiagen DNAEasy Blood and Tissue Kit (Cat. ID 69506, Valencia, CA). PCR was performed using AmpliTaq (Thermo-Scientific, Waltham, MA) on 200ng of isolated gDNA, with amplification parameters optimized for an amplicon size of 605bp with forward primer 5’-CTGGACGGCGACGTAAACGGC-3’ and reverse primer, 5’-ACCATGTGATCGCGCTTCTCG-3’. Amplicon size was verified on 1% agarose gel and PCR samples were cleaned up using the QIAquick PCR purification kit (Qiagen, Hilden, Germany). After purification, 300ng of PCR sample was combined with Buffer 3.1 and 25pmols or 50pmols of RNP complex. The mix was incubated for 40 minutes at 37°C then 1 microliter of proteinase K was added to the mix and incubated for 15 minutes. Samples were loaded along with NEB 2-log DNA ladder (NEB, Ipswich, MA) and analyzed on a 2% TBE agarose gel.

Cellular gDNA was isolated from pellets of 1–2 x 10^6^ K562 cells using the Qiagen DNEasy Blood and Tissue Kit (Cat. ID 69506, Valencia, CA). PCR was performed using Phusion High-Fidelity PCR Master Mix with HF Buffer (Thermo-Scientific, Waltham, MA) on isolated gDNA, with amplification parameters optimized for an amplicon size of 345bp with forward primer 5’- TCCTAAGCCAGTGCCAGAAGAG -3’ and reverse Primer 5’- CTATTGGTCTCCTTAAACCT -3'. Amplicon size was verified on 1% agarose gel. PCR samples were cleaned up using the QIAquick PCR purification kit (Qiagen, Hilden, Germany) and treated with DdeI restriction enzyme (NEB, Ipswich, MA) following the manufacturer’s protocol or RNP following the method described above. Digested samples were loaded along with NEB 2-log DNA ladder (NEB, Ipswich, MA) and analyzed on a 2% TBE agarose gel.

### DNA Sequence Analysis

Synchronized and released HCT 116–19 cells were harvested and electroporated at a concentration of 105 cells/100 microliters with RNP complex at 100pmols and 72NT ODN at 2.0 micromolar. Following electroporation, cells were placed in 6-well plates and allowed to recover for 72 hours. Cells were then individually sorted by a BD FACSAria II sorter - 488nm (100mw) (BD Biosciences, San Jose, CA) for eGFP+/- into 96-well plates. Cells were expanded over 6 weeks and harvested. Cellular gDNA was isolated using Qiagen DNEasy Blood and Tissue Kit (Cat. ID 69506, Valencia, CA) and the region surrounding the target base was amplified via PCR (718bp, forward primer 5’-ATGGTGAGCAAGGGCGAGGA-3’ and reverse primer 5’-ACTTGTACAGCTCGTCCATGC-3’). Samples were submitted to Eton Bio Incorporated (Union, NJ) for sequencing analysis.
